# Differences in circulating fatty acid-binding protein 4 concentration in the venous and capillary blood immediately after acute exercise

**DOI:** 10.1186/s40101-021-00255-z

**Published:** 2021-02-10

**Authors:** Shigeharu Numao, Ryota Uchida, Takashi Kurosaki, Masaki Nakagaichi

**Affiliations:** Department of Sports and Life Sciences, National Institute of Fitness and Sports in Kanoya, 1 Shiromizu, Kanoya, Kagoshima, 891-2393 Japan

**Keywords:** Acute exercise, Bland–Altman plot, Blood parameter, Adipokine

## Abstract

**Background:**

Circulating fatty acid-binding protein 4 (FABP4) is a marker for various diseases. It would be highly useful to have simple and less invasive techniques for the assessment of FABP4 concentrations in the clinical research setting. The purpose of the present study was to assess the concordance of circulating FABP4 concentrations in venous and capillary blood both at rest and immediately after acute exercise in healthy young males.

**Results:**

Thirty-eight healthy young male adults aged from 19 to 25 years (mean age, 20.8 ± 1.2 years) were recruited. Paired blood samples were taken from the cubital vein (venous) and fingertip (capillary) blood at rest (resting state) and immediately after incremental exercise (exercising state). Blood samples were analyzed to determine the circulating FABP4 concentration using an enzyme-linked immunosorbent assay. Pearson’s correlation coefficients for circulating FABP4 concentrations between venous and capillary blood samples indicated a strong positive correlation in both the resting and exercising state (resting state: *r* = 0.982, exercising state: *r* = 0.989, both *p* < 0.001). The mean FABP4 concentration was similar between venous and capillary blood in the resting state (*p* = 0.178), whereas it was significantly higher in capillary blood than in venous blood in the exercising state (*p* < 0.001). Furthermore, Bland–Altman plots showed a non-significant bias (− 0.07 ± 0.61 ng/mL, *p* = 0.453) in the resting state, whereas a significant bias (− 0.45 ± 0.61 ng/mL, *p* < 0.001) was observed in the exercising state.

**Conclusions:**

These results indicate that capillary blood sampling can slightly overestimate circulating FABP4 concentrations under a physiologically dynamic state. However, the association between the venous and capillary blood in terms of FABP4 concentration was very strong, suggesting that capillary blood sampling can detect changes in FABP4 concentration in both physiologically steady and dynamic states.

**Supplementary Information:**

The online version contains supplementary material available at 10.1186/s40101-021-00255-z.

## Introduction

Fatty acid-binding protein 4 (FABP4), also known as adipocyte FABP or adipose protein 2, is one of the fatty acid-binding proteins. The FABPs are abundantly expressed 14–15 kDa cytosolic lipid chaperones that regulate lipid trafficking and responses in cells [1]. FABP4 is highly expressed in adipocytes and macrophages [2–4] and is primarily secreted from adipocytes into the blood [5, 6].

Circulating FABP4 concentration is known as a biomarker for several disorders; increased circulating FABP4 concentrations are associated with the increased risk of various diseases, such as atherosclerosis [7, 8], insulin resistance [9], type 2 diabetes [10], hypertension [9, 11], dyslipidemia [9, 12], cardiovascular diseases [13, 14], and cancer [15]. Notably, it has been also recently reported to be a potential biomarker for autism spectrum disorder in children [16]. Thus, circulating FABP4 concentration is increasingly useful as a biomarker of disorders. Therefore, to broaden the use of circulating FABP4 as a biomarker in clinical research settings, simpler and less invasive techniques are required to determine its concentrations.

To date, venous blood sampling is the most widely used method for assessing circulating FABP4 concentrations. However, venous blood sampling is relatively invasive, requires a trained phlebotomist, generates biological waste, creates participant discomfort, and interrupts physical activity (exercise) [17, 18]. Therefore, capillary blood sampling from the fingertip has been used as a simple alternative method to obtain blood samples. Capillary blood sampling is minimally invasive, can be performed by non-specialist staff, helps avoid excessively restricting physical activities, and reduces the discomfort of participants. Therefore, it is an efficient and convenient method for assessing circulating FABP4 concentration in clinical research settings.

In our previous study, we compared circulating FABP4 concentrations between venous and capillary blood [19]; the concentrations were almost identical between venous and capillary blood under a physiologically steady state (resting state). However, we did not investigate the concentrations under a physiologically dynamic state, such as during exercise. A recent study reported the probability that circulating FABP4 concentrations serve as a biomarker for estimating catecholamines response and lipolysis by adrenergic stimulation in vivo [20] as well as for predicting a risk of various disorders. This suggests that the circulating FABP4 concentration will be applied as a biomarker under a physiologically dynamic state when adrenergic stimulation enhanced. This necessitates the analyses of circulating FABP4 concentration under various states to expand its application as a biomarker in clinical research setting. Besides, whether circulating FABP4 concentrations are identical between venous and capillary blood under a physiologically dynamic state is unclear. Thus, to establish capillary blood sampling as a viable alternative for blood sampling in clinical research, it is necessary to estimate the difference in circulating FABP4 concentrations between venous and capillary blood, even under a physiologically dynamic state.

The purpose of the present study was to assess the concordance of circulating FABP4 concentrations between venous and capillary blood under both a physiologically steady (i.e., resting state) and dynamic state (i.e., immediately after exercise [exercising state]) in healthy young males. We hypothesized that circulating FABP4 concentrations in the capillary blood would accurately and precisely reflect those in venous blood under both physiological states.

## Materials and methods

### Participants

Thirty-eight healthy young males (mean age, 20.8 ± 1.2 years) participated in the present study (Table 1). All participants were undergraduate and graduate students who responded to our advertisement featuring the experiment (experimental protocol, period, and measurement parameters) and reward details. The exclusion criteria were as follows: applications 1) who were currently unhealthy (i.e., currently receiving medical treatments); 2) who had a history of metabolic disorders, cardiovascular diseases, or cancer in the past; and 3) who were smoking or currently taking some medications or supplements. This information was confirmed via interview. The purpose, design, and risks involved in the present study were explained to all participants, and each provided written informed consent. The study conformed to the principles outlined in the Declaration of Helsinki and was approved by the Ethics Committee of the National Institute of Fitness and Sports in Kanoya (approval number: 11-94).

**Table 1:** Physical characteristics of all participants (*n* = 38)

	Mean	±	Standard deviation
Age (years)	20.8	±	1.2
Height (cm)	172.8	±	6.3
Weight (kg)	72.4	±	12.0
Body mass index (kg/m^2^)	24.2	±	3.6
%fat (%)	17.3	±	6.2
Fat mass (kg)	12.9	±	6.5
Fat-free mass (kg)	59.5	±	8.0
Skeletal muscle mass (kg)	56.6	±	7.7

### Study procedure

Participants completed all study components and measurements during a single session in the following order: (1) general health interview (current and previous medical history), (2) anthropometry and body composition measurements, (3) venous and capillary blood sampling at rest, (4) acute incremental exercise, and (5) venous and capillary blood sampling immediately after acute exercise. Participants were instructed to refrain from strenuous exercise for at least 24 h and from the consumption of meals except for water for at least 12 h before the study visit.

### Anthropometry and body composition measures

For each participant, the height was measured to the nearest 0.1 cm using a stadiometer. Weight, body-fat percentage (%fat), fat-mass (FM), fat-free mass (FFM), and skeletal muscle mass (SMM) were measured to the nearest 0.1 kg using the dual-frequency impedance method (RD-800; Tanita Corporation, Tokyo, Japan). Body mass index (BMI) was calculated as weight (in kilograms) divided by height (in meters) squared.

### Exercise procedure

After blood sampling at rest, an exercise test was performed on a cycle ergometer (Aerobike 75XLIII, Konami Sports Life, Kanagawa, Japan). The exercise protocol, after a brief individual warm-up period, consisted of a 30-watt increase every 2 min at 60 watts until 6 min and thereafter a 30-watt increase every 1 min until exhaustion.

### Blood sampling and analysis

Paired blood samples were collected from the cubital vein and the fingertip at rest and after acute exercise. Blood samples were collected in the following order: venous blood and then capillary blood. The interval between the venous and capillary blood sampling was less than 3 min. Venous and capillary blood samplings were performed using a single-use blood-collecting holder with the needle and a single-use lancing device, respectively. Venous blood samples were collected in a 9-mL tube containing a blood coagulation accelerator (Venoject ΙΙ, Terumo Corporation, Tokyo, Japan). Capillary blood samples were collected in two 150-μL microtubes (‘Kantan’ tube, Eiken Chemical Corporation, Tokyo, Japan). Approximately, 300 μL of the capillary blood was collected in the microtubes within approximately 2 min. Venous blood samples were centrifuged at 3000*g* for 10 min at 25 °C 30 min after collection; capillary blood samples were centrifuged at 2000*g* for 5 min at room temperature 30 min after collection. Following centrifugation, the serum obtained from each sample was transferred to plastic tubes and immediately stored at − 80 °C until further analysis.

Circulating FABP4 concentrations were analyzed using an enzyme-linked immunosorbent assay (ELISA) (R&D system Inc., Minneapolis, USA; intra-assay coefficient of variation: < 5.8%). The ELISA procedure was performed according to the manufacturer’s instructions. Each sample was analyzed in duplicate. Samples from each participant were analyzed in the same run to eliminate inter-assay variation.

### Statistical analysis

The data from four (resting state data) and five (exercising state data) participants were excluded from statistical analysis, because their blood samples were hemolyzed. All data are presented as the mean ± standard deviation and were tested for normality using the Kolmogorov–Smirnov test. As normality was not assumed, FABP4 concentrations were log-transformed to estimate the difference and perform correlation analyses. The sample size was calculated to detect a large effect (Cohen’s *d* = 0.80 and *r* = 0.50). A sample size of 15–26 participants would be required to have approximately 80% power to detect a large effect with 0.05 significance. A two-tailed paired *t* test was used to compare the differences between the venous and capillary blood in circulating FABP4 concentration in each resting and exercising state. Bland–Altman plots with 95% limits of agreement (± 1.96 × SD) [21] and Pearson’s correlation coefficients were used to assess the correlation between the venous and capillary blood samples in each resting and exercising state. A regression analysis was performed—with the difference in circulating FABP4 concentrations between the venous and capillary blood samples as the dependent variable and the mean venous and capillary FABP4 concentrations as the independent variable—to evaluate proportional bias in the Bland–Altman plots. The effect size (ES) was calculated using Cohen’s *d* for the comparison of circulating FABP4 concentration in the venous and capillary blood. The reference values for the ES were as follows: Cohen’s *d*, small: ≥ 0.20, medium: ≥ 0.50, or large: ≥ 0.80, and *r*, small: ≥ 0.10, medium: ≥ 0.30, or large: ≥ 0.50. Statistical analyses were performed using the SPSS Statistics version 24 (IBM Corporation, NY, USA). Statistical significance was set at *p* < 0.05.

## Results

### Resting state

**Fig. 1:**
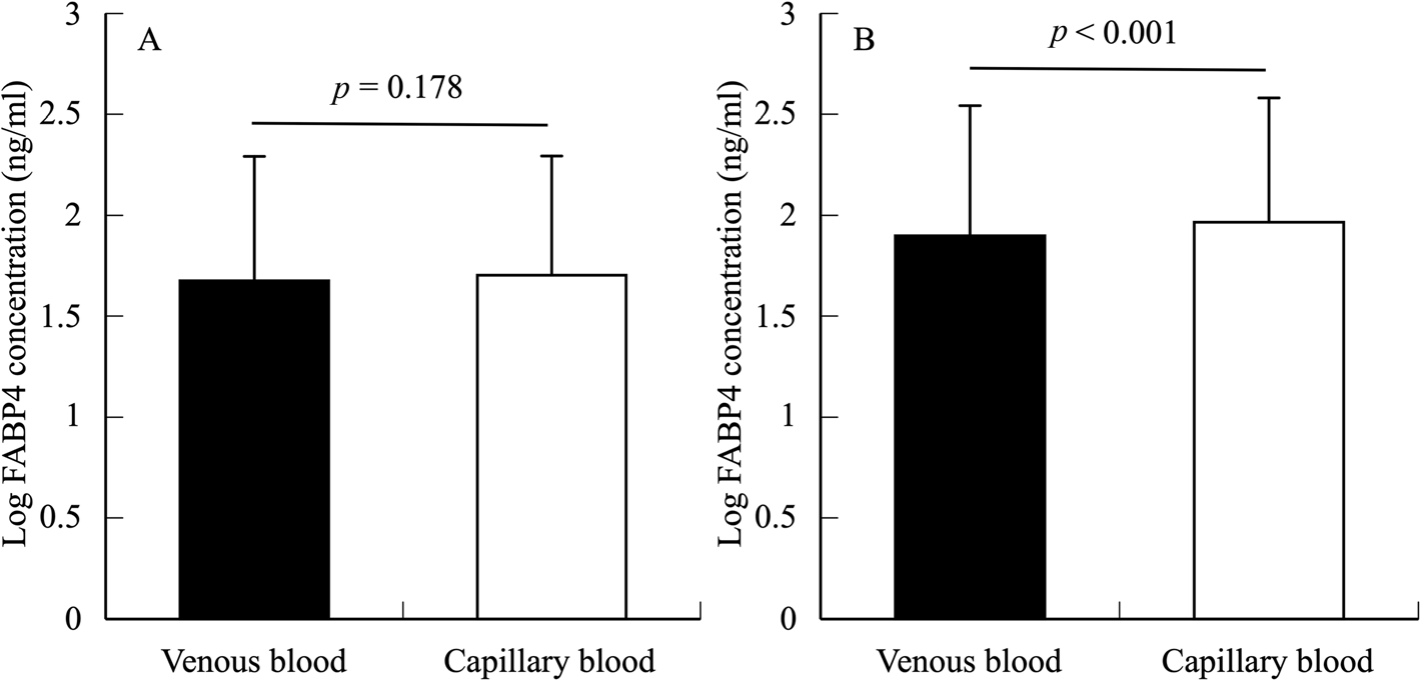
Difference in circulating FABP4 concentration between the venous and capillary blood in the resting (**a**) and exercising state (**b**). Data are presented as the mean ± standard deviation. There was no significant difference in circulating FABP4 concentration between the venous and capillary blood in the resting state (*p* = 0.178). A significant difference in circulating FABP4 concentration between the venous and capillary blood was observed in the exercising state (*p* < 0.001)

**Fig. 2:**
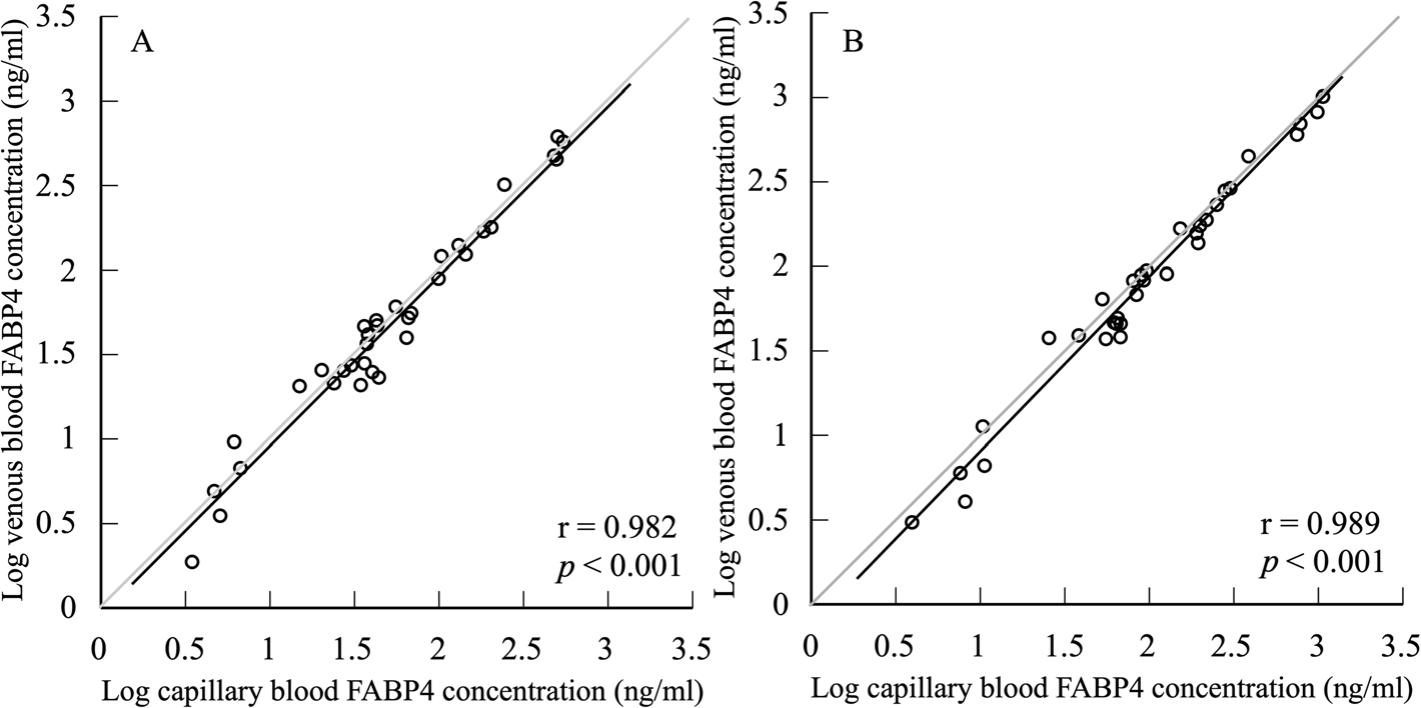
Scatter plots of circulating FABP4 concentrations between the venous and capillary blood in the resting (**a**) and exercising state (**b**). Dark line indicates an identical line. Pearson’s correlation coefficients, the resting state: *r* = 0.982 (*p* < 0.001), and the exercising state: *r* = 0.989 (*p* < 0.001)

**Fig. 3:**
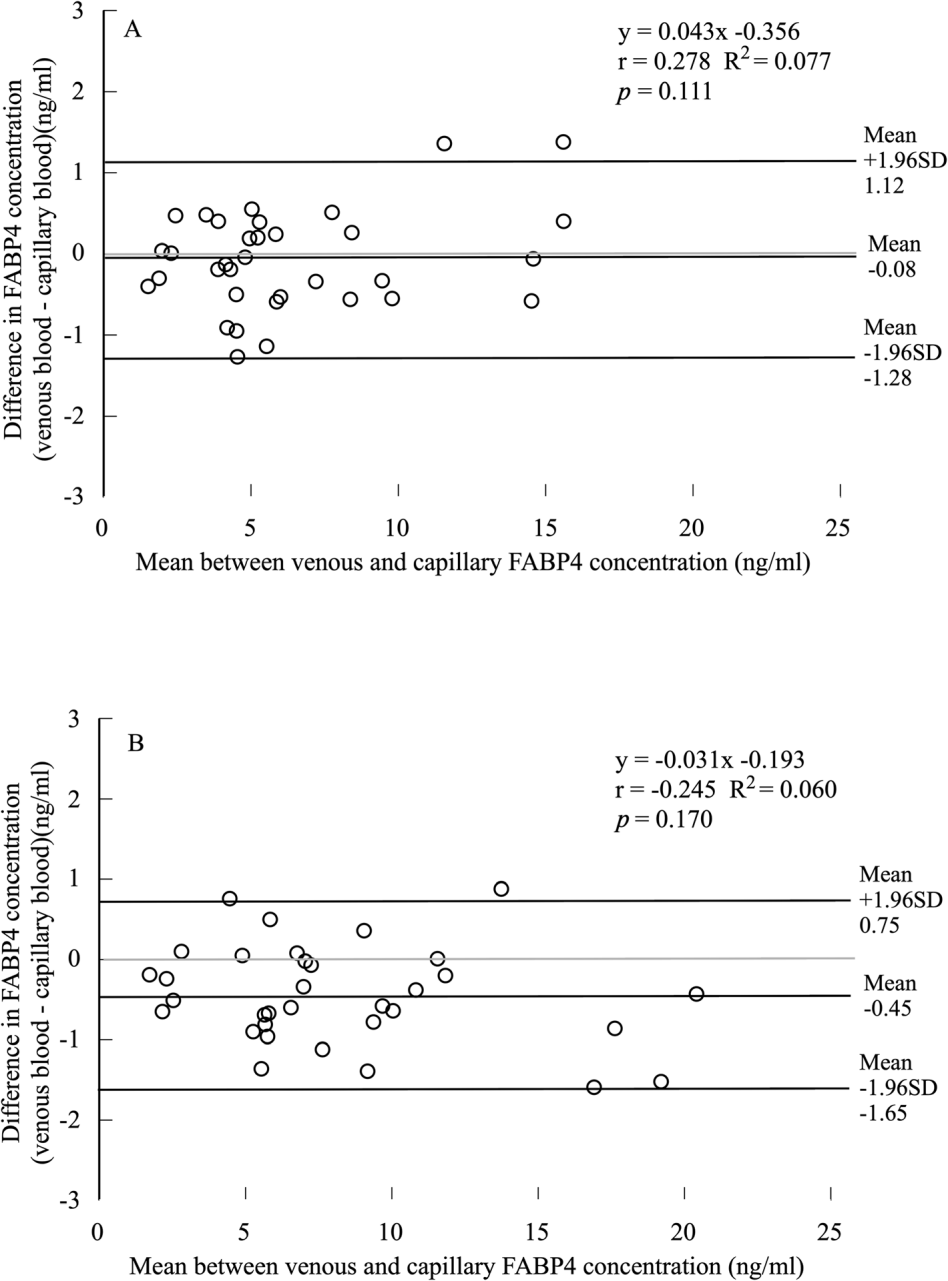
Bland–Altman plots showing the difference against the average circulating FABP4 concentrations between the venous and capillary blood in the resting (**a**) and exercising state (**b**). Solid lines represent the mean bias and the 95% limits of agreement. There was no significant bias in circulating FABP4 concentrations between the venous and capillary blood in the resting state (*p* = 0.453). A significant bias in circulating FABP4 concentrations between the venous and capillary blood was observed in the exercising state (*p* < 0.001). There was no correlation between the difference and average circulating FABP4 concentrations between the venous and capillary blood in both resting (*p* = 0.111) and exercising state (*p* = 0.170)

The data from 34 participants were included in the final analyses. The log circulating FABP4 concentration did not significantly differ between the venous and capillary blood (venous: 1.68 ± 0.62 ng/ml, capillary: 1.70 ± 0.59 ng/ml, *t* = −1.376, degree of freedom (*df*) = 33, *p* = 0.178, 95% confidence interval (CI): −0.068–0.013, ES: 0.03) (Fig. 1a). The Pearson’s correlation coefficient for log circulating FABP4 concentrations between the venous and capillary blood was significantly large (*r* = 0.982, *p* < 0.001) (Fig. 2a). The Bland–Altman plots showed a non-significant bias (−0.08 ± 0.61 ng/mL, *p* = 0.453, 95%CI: −0.292–0.133) in circulating FABP4 concentrations between venous and capillary blood (Fig. 3a). The regression analysis showed that the estimated regression equation was not significant (*y* = 0.043*x*–0.356, *p* = 0.111, estimated standard error (SEE) = 0.594, 95%CI: −0.010–0.096). The 95% limits of agreement ranged from − 1.28 to 1.12 ng/mL. Two outliers were observed in the Bland–Altman plot, but 94.1% of plots were included in the 95% limits of agreement.

### Exercising state

The data from 33 participants were included in the final analyses. In contrast to the resting state, the log circulating FABP4 concentration in the capillary blood was significantly higher than that in the venous blood (venous: 1.89 ± 0.64 ng/ml, capillary: 1.97 ± 0.61 ng/ml, *t* = -3.953, *df* = 32, *p* < 0.001, 95%CI: −0.120 – −0.033) (Fig. 1b). However, the ES value was small (0.11). The Pearson’s correlation coefficient for log circulating FABP4 concentrations between venous and capillary blood was significantly large (*r* = 0.989, *p* < 0.001) (Fig. 2b). The Bland–Altman plots indicated a significant bias (−0.45 ± 0.61 ng/mL, *p* < 0.001, 95%CI: −0.666 – −0.230) in circulating FABP4 concentrations between venous and capillary blood (Fig. 3b). The regression analysis showed that the estimated regression equation was not significant (*y* = −0.031*x*–0.193, *p* = 0.170, SEE = 0.60, 95%CI: −0.076–0.014). The 95% limits of agreement ranged from − 1.65 to 0.75 ng/mL. Two outliers were observed in the Bland–Altman plots; however, 93.9% of plots were included in the 95% limits of agreement.

## Discussion

The present study compared the circulating FABP4 concentration between the venous and capillary blood in each resting and exercising states in young healthy males. We hypothesized that circulating FABP4 concentrations in capillary blood would accurately and precisely reflect those in the venous blood under both the physiologically states. However, our results only partially supported our hypothesis. We observed a strong correlation between the venous and capillary blood in the resting and exercising states. While circulating FABP4 concentrations were similar between the venous and capillary blood in the resting state, the FABP4 concentrations were significantly higher in the capillary blood than in the venous blood in the exercising state. Additionally, despite a non-significant bias of FABP4 concentrations in the resting state, a significant bias was found in the exercising state. These findings indicate that capillary blood sampling can slightly overestimate the circulating FABP4 concentration in the exercising state. However, there was a very strong association between FABP4 concentrations in the venous and capillary blood, suggesting that capillary blood sampling can detect changes in FABP4 concentration in both resting and exercising states.

Our findings in the resting state support our previously reported results [19]. However, the characteristics of participants in the present study slightly differed from that of our previous study [19]. In short, the BMI, FM, %fat, FFM, SMM, and circulating FABP4 concentration in resting state were higher in the present study compared to our previous study [19]. Circulating FABP4 concentration is known to be correlated with BMI, FM, and %fat [9, 19, 22]; this has been confirmed in the present study (Table S1). Meanwhile, circulating FABP4 concentrations were not significantly correlated with FFM and SMM (Table S1), suggesting that the influence of FFM and SMM on circulating FABP4 concentration is small. Because %fat or FM was higher in the present study, FABP4 concentrations measured in the resting state (a non-log-transformed FABP4 concentration, venous: 6.4 ± 4.0 ng/ml, capillary: 6.5 ± 3.9 ng/ml) was higher in the present study compared to that in our previous study (venous: 4.3 ± 2.4 ng/ml, capillary: 4.5 ± 2.5 ng/ml) [19]. Nevertheless, we found no differences in FABP4 concentrations between the venous and capillary blood in the resting state, and the corresponding Bland–Altman plots indicated a non-significant negative and proportional bias. Additionally, the ES of the difference in the present study (ES = 0.03) was almost identical to that in our previous study (ES = 0.04). Although the range of the 95% limits of agreement is narrower in the present study, the data included in the 95% limits of agreement are similar to our previous study [19]. Taken together, these findings suggest that capillary blood sampling is a reliable alternative to venous blood sampling to assess circulating FABP4 concentrations under a physiologically steady state, even in healthy young males with relatively higher BMI and fat mass.

Capillary blood sampling is commonly used to measure blood metabolite concentrations (e.g., glucose, lipoprotein, and lactate concentration) in clinical settings [23–27]. Because the capillary blood is an intermediate between the arterial and venous blood, the metabolite concentrations in the capillary blood tend to be higher than that in venous blood [23–27]. In addition, this difference in metabolite concentrations between the capillary and venous blood is more marked in a state of increased blood metabolite concentrations (i.e., the postprandial and the exercising state) [23, 27–33]. This has also been observed in cytokine concentrations [34]. Circulating interleukin-6 concentration is higher in capillary than venous blood at rest, and the difference is greater after acute high-intensity exercise [34]. In the present study, the FABP4 concentration was elevated in the exercising state in both the capillary and venous blood compared with the resting state. This suggests that the secretion of FABP4 into the circulation is accelerated during exercise. Similar to the demonstrated difference in blood metabolite concentrations between the capillary and venous blood under a physiologically dynamic state [23, 27–34], the acute increase in FABP4 concentration may lead to the difference and negative bias in FABP4 concentrations between the capillary and venous blood in the exercising state. Thus, capillary blood sampling may slightly overestimate FABP4 concentrations under a physiologically dynamic state, compared with venous blood sampling.

While the FABP4 concentration in the capillary blood was higher than that in venous blood in the exercising state, the absolute difference in FABP4 concentration between the capillary and venous blood was slight, and the ES was small. Additionally, the correlation in FABP4 concentrations between the capillary and venous blood was very strong. In the exercising state, Bland–Altman plots showed a non-significant proportional bias. It also indicated that 93.9% of the plot was included within the 95% limits of agreement. Although the bias and the 95% limits of agreement must be interpreted from a clinical perspective, few studies have reported the circulating FABP4 concentration during exercise. Only one study reported a change in circulating FABP4 concentrations after approximately 10-min constant-load aerobic exercise [20]. Circulating FABP4 concentrations were observed to increase from approximately 11 to 19 ng/mL (~ 8 ng/mL difference) during the exercise. This change in circulating FABP4 concentration during the exercise is far beyond the bias and the 95% limits of agreement range (~ 2.4 ng/mL) of the present study. This would suggest that, given that the change in FABP4 concentration during aerobic exercise is relatively large, capillary blood sampling is an acceptable method for the assessment of changes in circulating FABP4 concentrations during acute aerobic exercise. However, capillary blood sampling is probably not reliable enough to detect the small changes in FABP4 concentrations, thus limiting its applicability in clinical research.

The present study has several limitations. First, the participants were young healthy males; therefore, our findings may not be applicable to females, obese individuals, and older adults with or without diseases, and cancer patients. Circulating FABP4 concentrations are higher in females and patients with metabolic disorders, cardiovascular diseases, and cancer than in young healthy males [7–15]. It is necessary to consider the correlation of circulating FABP4 concentrations between the venous and capillary blood in a population with higher baseline circulating FABP4 concentrations. Second, sampling of the venous and capillary blood was performed under both physiologically steady and dynamic conditions. However, the dynamic state modelled in this study acutely increases FABP4 concentrations in the blood. Therefore, our findings may not be applicable to dynamic states that acutely decrease FABP4 concentration in the blood, such as after food consumption [6]. Thus, further studies in other populations and situations are warranted to ascertain the generalizability of our results.

In conclusion, the findings of the present study demonstrate that the capillary blood sampling is a viable alternative to venous blood sampling under a physiologically steady state. While capillary blood sampling, compared with venous blood sampling, slightly overestimates FABP4 concentrations under a physiologically dynamic state, the method is suitable for tracking changes in circulating FABP4 concentration in healthy young males.

## Supplementary Information

**Additional file 1.**

## Data Availability

All data generated or analyzed during this study are included in this published article.

## Abbreviations

FABP4: Fatty acid-binding protein 4
FABPs: Fatty acid-binding proteins
%fat: Percentage of body fat
FM: Fat mass
FFM: Fat-free mass
SSM: Skeletal muscle mass
BMI: Body mass index
SD: Standard deviation
ES: Effect size
CI: Confidence interval
SEE: Estimated standard error
