# Supplementary material for: Differences in circulating fatty acid-binding protein 4 concentration in the venous and capillary blood immediately after acute exercise
Source: J Physiol Anthropol. 2021 Feb 10;40:5. doi: 10.1186/s40101-021-00255-z (PMC7876805; doi:10.1186/s40101-021-00255-z)
Supplement: Supplementary file 1 — Additional file 1. [file 40101_2021_255_MOESM1_ESM.docx]

**Supplementary Material**

| **Table S1**. Pearson correlation coefficients between body composition and fatty acid binding protein 4 concentrations. | | | | | | | | | | | |
| --- | --- | --- | --- | --- | --- | --- | --- | --- | --- | --- | --- |
|  | Venous | |  | Capillary | |  | Venous  (log-transformed) | |  | Capillary  (log-transformed) | |
|  | R | E |  | R | E |  | R | E |  | R | E |
| Body mass index | **0.543** | **0.629** |  | **0.493** | **0.583** |  | **0.447** | **0.496** |  | **0.411** | **0.468** |
| *p* | < 0.001 | < 0.001 |  | < 0.001 | < 0.001 |  | < 0.001 | < 0.001 |  | 0.016 | < 0.001 |
| Percentage of fat | **0.597** | **0.633** |  | **0.569** | **0.623** |  | **0.486** | **0.495** |  | **0.454** | **0.490** |
| *p* | < 0.001 | < 0.001 |  | < 0.001 | < 0.001 |  | < 0.001 | < 0.001 |  | < 0.001 | < 0.001 |
| Fat mass | **0.582** | **0.637** |  | **0.535** | **0.605** |  | **0.473** | **0.494** |  | **0.436** | **0.479** |
| *p* | < 0.001 | < 0.001 |  | < 0.001 | < 0.001 |  | < 0.001 | < 0.001 |  | < 0.001 | < 0.001 |
| Fat-free mass | 0.069 | 0.140 |  | 0.029 | 0.082 |  | 0.081 | 0.140 |  | 0.065 | 0.106 |
| *p* | 0.697 | 0.437 |  | 0.869 | 0.649 |  | 0.648 | 0.437 |  | 0.716 | 0.558 |
| Skeletal muscle mass | 0.065 | 0.129 |  | 0.023 | 0.072 |  | 0.083 | 0.134 |  | 0.065 | 0.106 |
| *p* | 0.713 | 0.473 |  | 0.898 | 0.689 |  | 0.640 | 0.458 |  | 0.725 | 0.586 |
| FABP4, fatty acid binding protein 4; R, resting state; E, exercising state. | | | | | |  |  |  |  |  |  |
